# Omega-3 and Omega-6 Polyunsaturated Fatty Acid Levels and Correlations with Symptoms in Children with Attention Deficit Hyperactivity Disorder, Autistic Spectrum Disorder and Typically Developing Controls

**DOI:** 10.1371/journal.pone.0156432

**Published:** 2016-05-27

**Authors:** Natalie Parletta, Theophile Niyonsenga, Jacques Duff

**Affiliations:** 1 Centre for Population Health Research, School of Health Sciences,University of South Australia, Adelaide, Australia; 2 Behavioural Neurotherapy Clinic, Doncaster, Victoria, Australia; 3 Australian Autism ADHD Foundation, Victoria, Australia; Torrey Pines Institute for Molecular Studies, UNITED STATES

## Abstract

**Background:**

There is evidence that children with Attention Deficit Hyperactivity Disorder (ADHD) and Autistic Spectrum Disorder (ASD) have lower omega-3 polyunsaturated fatty acid (n-3 PUFA) levels compared with controls and conflicting evidence regarding omega-6 (n-6) PUFA levels.

**Objectives:**

This study investigated whether erythrocyte n-3 PUFAs eicosapentaenoic acid (EPA) and docosahexaenoic acid (DHA) were lower and n-6 PUFA arachidonic acid (AA) higher in children with ADHD, ASD and controls, and whether lower n-3 and higher n-6 PUFAs correlated with poorer scores on the Australian Twin Behaviour Rating Scale (ATBRS; ADHD symptoms) and Test of Variable Attention (TOVA) in children with ADHD, and Childhood Autism Rating Scale (CARS) in children with ASD.

**Methods:**

Assessments and blood samples of 565 children aged 3–17 years with ADHD (*n* = 401), ASD (*n* = 85) or controls (*n* = 79) were analysed. One-way ANOVAs with Tukey’s post-hoc analysis investigated differences in PUFA levels between groups and Pearson’s correlations investigated correlations between PUFA levels and ATBRS, TOVA and CARS scores.

**Results:**

Children with ADHD and ASD had lower DHA, EPA and AA, higher AA/EPA ratio and lower n-3/n-6 than controls (*P*<0.001 except AA between ADHD and controls: *P* = 0.047). Children with ASD had lower DHA, EPA and AA than children with ADHD (*P*<0.001 for all comparisons). ATBRS scores correlated negatively with EPA (*r* = -.294, *P*<0.001), DHA (*r* = -.424, *P*<0.001), n-3/n-6 (*r* = -.477, *P*<0.001) and positively with AA/EPA (*r* = .222, *P* <.01). TOVA scores correlated positively with DHA (*r* = .610, *P*<0.001), EPA (r = .418, *P*<0.001) AA (*r* = .199, *P*<0.001), and n-3/n-6 (*r* = .509, *P*<0.001) and negatively with AA/EPA (*r* = -.243, *P*<0.001). CARS scores correlated significantly with DHA (*r* = .328, *P* = 0.002), EPA (*r* = -.225, *P* = 0.038) and AA (*r* = .251, *P* = 0.021).

**Conclusions:**

Children with ADHD and ASD had low levels of EPA, DHA and AA and high ratio of n-6/n-3 PUFAs and these correlated significantly with symptoms. Future research should further investigate abnormal fatty acid metabolism in these disorders.

## Introduction

Attention deficit hyperactivity disorder (ADHD) and autistic spectrum disorder (ASD) are neurodevelopmental disorders that impact quality of life and have significant psychiatric comorbidities [1, 2]. ADHD is estimated to affect 5.29% of children globally [3]. The 2014 US National Health Statistics Report indicated an overall 2.24% prevalence of ASD, nearly doubled from 1.25% annual prevalence identified by 2011–13 data [4]. Although the underlying etiology is unknown, these disorders have a genetic component [5, 6] which may be exacerbated by environmental factors including industrial and environmental chemicals [7, 8], a western style diet [9–11], and perinatal influences [12]. Recent evidence suggests that the gut microbiota [13] and bowel dysfunction may play contributing roles.

ADHD is characterized by age-inappropriate levels of inattention, impulsivity and hyperactivity to a degree that impacts on day-to-day functioning. Children with ADHD are often restless and can have difficulties following instructions. Symptoms persist across different settings (e.g. school and home) [14]. ASD is a pervasive developmental disorder (PDD) that includes the former diagnostic labels of autistic disorder, Asperger’s disorder, childhood integrative disorder, and PDD not otherwise specified. ASD is characterised by communication deficits and difficulty with social interactions. Symptoms may include overdependence on routines, restricted and/or repetitive behaviours and interests, and hyper- or hypo-sensitivity to the surrounding environment [14].

Medications are the most common treatment approach for neurodevelopmental disorders. Often they can be efficacious in treating symptoms but can have unacceptable adverse side effects in the short and long term [15–19]. There has been growing interest in the role of nutrition in ADHD and/or ASD, with increasing evidence to support a possible role of nutritional factors in the development, treatment and prevention of these neurodevelopmental disorders—particularly omega-3 polyunsaturated fatty acids (n-3 PUFAs) [20–24].

The central nervous system is rich in n-3 and omega 6 (n-6) PUFAs [25], in particular, the n-3 PUFA docosahexaenoic acid (DHA) and to a lesser extent n-6 PUFA arachidonic acid (AA). DHA is highly active in the retina and in synapses, where it modulates the synthesis, transport and release of neurotransmitters. DHA also plays a primary role in neurite growth, membrane fluidity, neurotransmission, endothelial function, neuronal survival and attenuating neurodegeneration [25]. PUFAs cannot be manufactured by humans and must be obtained through dietary sources [26]. However dietary intake of n-3 PUFAs has declined and the estimated ratio of n-6 to n-3 PUFAs has risen from 1:1 in traditional diets to around 16:1 in western diets. This is of concern as dietary PUFAs from plant sources compete for the same enzymes for elongation and desaturation to long-chain PUFAs. Hence excess n-6 PUFAs may displace n-3 PUFAs in cellular membranes. The altered ratio may result in increased inflammation, thrombosis and vasoconstriction (hence blood flow) due to opposing properties of n-6 and n-3 derived eicosaoids from arachidonic acid (AA) and eicosapentaenoic acid (EPA), respectively [27]. This has implications for mental illness which has inflammation and reduced cerebral blood flow as part of its pathology [28].

A recent meta-analysis of 9 studies (N = 586) found that children with ADHD had lower overall blood n-3 PUFA levels than controls, in particular DHA [29]. Some, but not all studies also reported higher levels of n-6 PUFA arachidonic acid (AA) and/or higher ratio of n-6 to n-3 PUFA [30–32] and one conversely reported lower AA in children with ADHD [31]. Despite some methodological issues and inconsistencies, research suggests that children with ADHD benefit from supplementation with n-3 PUFAs [24]. Part of this benefit may be attributable to lowering the n-6/n-3 ratio. One study in children with ADHD showed consistent associations between increased n-3 PUFAs (particularly DHA) decreased AA, n-6 PUFAs and n-6/n-3 ratio and improved cognitive and behavioural outcomes [33].

There is also evidence for lower n-3 PUFA levels in children with autism although results are conflicting [34–38]. This study aimed to compare erythrocyte PUFA levels in children with ADHD, ASD and typically developing controls, and to investigate correlations between PUFA levels and respective symptoms. We hypothesised that children with ADHD and ASD would have lower EPA and DHA, higher AA, higher AA/EPA ratio and lower n-3/n-6 PUFA ratio than controls and that these PUFAs would correlate with poorer cognitive and behavioural symptoms.

## Methods

### Participants

The study included *N* = 565 children with ADHD (*n* = 401) or ASD (*n* = 85) and typically developing controls (*n* = 79) aged between 3 and 17 years (M = 8.42 ± 3.53). Details on age and gender broken down by group are provided in Table 1. Children with ASD were significantly younger than those with ADHD (*P* < 0.001) and controls (*P* < 0.001) while those with ADHD and controls did not differ (*P* = 0.132). There were no significant differences in gender between the groups (*P* = 0.667–0.885). Many of the children with ADHD and none of the children with ASD were on stimulant medication.

**Table 1:** Age, gender and test scores of participants by group, *N* = 565.

*Group*	*ADHD (n = 401)*	*ASD (n = 85)*	*Controls (n = 79)*	*P*
*Age*: *M ± SD*	*9*.*10 ± 3*.*58*	*5*.*31 ± 2*.*12*	*8*.*32 ± 2*.*53*	*<0*.*001*^a^
*Gender*: *% boys*	*79*.*6*	*80*.*0*	*77*.*2*	*0*.*883*
*Tests of Variable Attention*: *M ± SD*	*-3*.*78 ± 3*.*28*	*N/A*	*3*.*85 ± 1*.*67*	*<0*.*001*
*ADHD scale*^b^	*42*.*43 ± 14*.*43*	*N/A*	*14*.*11*	*<0*.*001*
*CARS*	*N/A*	*40*.*71 ± 8*.*04*	*N/A*	*N/A*

^a^post-hoc analysis: ADHD children were significantly different from ASD children; ASD children were significantly different from controls.

^b^Australian Twin Behaviour Rating Scale (ATBRS)

### Procedure

**Fig 1:**
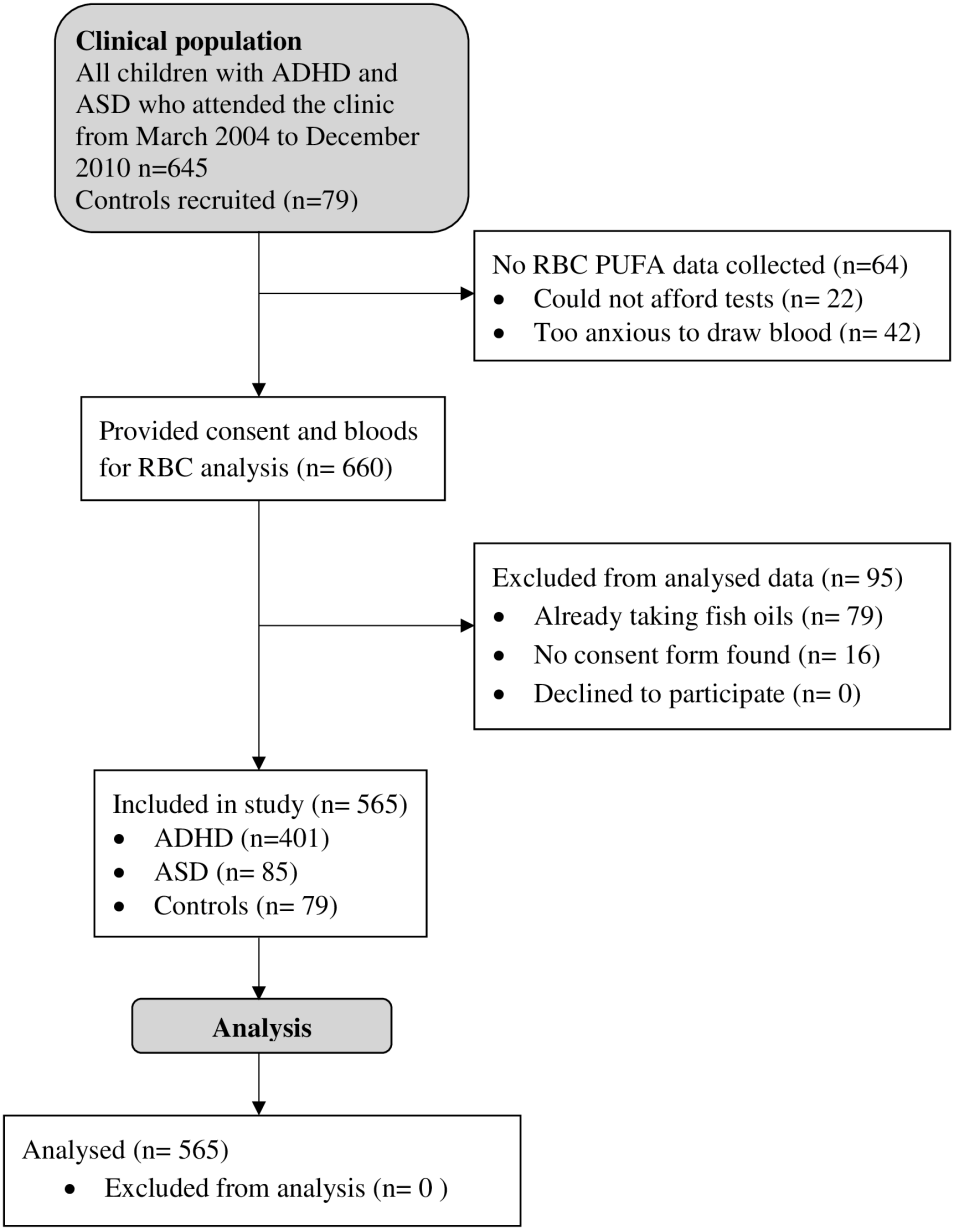
Flow of participants through the study.

The children with ADHD and ASD in this study presented at the Behavioural Neurotherapy Clinic (BNC) in Doncaster (Melbourne), Australia for treatment, between March 2004 and December 2010 (see Fig 1 for flowchart of children through the study). The children were diagnosed by a Paediatrician, most in conjunction with the help of a multidisciplinary team consisting of a Psychologist, an Occupational Therapist and a Speech Pathologist either at the BNC or externally. The control group of typically developing children were recruited from friends and relatives of families who attended the clinic and were offered free testing by Healthscope Pathology, who conducted the blood analyses, and Psychologists at the Behavioural Neurotherapy Clinic, who conducted the screening. Only participants whose parents or guardians gave written permission for their de-identified data to be used in future research were included in the study. Ethics approval for this study was provided by the Bellberry Human Research Ethics Committee (Protocol number 2015-08-592) in accordance with the National Health and Medical Research Council’s National Statement on Ethical Conduct in Human Research.

Participants who had taken any nutritional supplement during the previous year were excluded. As part of normal client intake at BNC, parents and/or guardians of each child are asked to sign an informed consent form informing them of their rights to privacy and the BNC’s obligations to mandatory reporting, and inviting them to either agree or disagree to have the de-identified results of psychological assessments and medical tests including results of blood tests stored and used for future research purposes. It was very rare, estimated at less than one in a hundred, that parents and/or guardians declined to participate. The data from those children and others for whom a signed consent form could not be found were excluded from the study. Also excluded were data from children with known genetic abnormalities, which may have confounded results. Inclusion criteria for the typically developing control group were no parent-reported academic or behavioural concerns, confirmed by a clinical interview with JD to ensure they did not meet the criteria for any childhood disorders from the Diagnostic and Statistical Manual of Mental Disorders (DSM-IV), and non-clinical scores on the ADHD scale that was used in the study (described below; results presented in Table 1).

### Tools

The following questionnaires and tests were undertaken by participating parents and children.

#### Tests of Variables of Attention (TOVA) version 7

The Tests of Variables of Attention are continuous performance tests used to assess attention and impulsivity and can be useful as part of a diagnostic assessment and for detecting treatment effects. They are non-verbal computerized tests with negligible practice effects [39] and have robust internal consistency [40]. Scores are derived for errors of omission, errors of commission, response time and response time variability. For more information about the test see Llorente et al [40]. A score of less than -1.80 is considered indicative of ADHD whereas scores >1 are considered ‘normal’. In this study the TOVA was administered to the ADHD and control samples.

#### Australian Twin Behaviour Rating Scale (ATBRS)

The ATBRS was developed and validated for a large-scale Australian twin study by Levy et al. [41]. The questions are based on the DSM version III-R of mental disorders. Twenty items are rated in terms of their occurrence on a scale of 0–3 whereby 0 = not at all and 3 = very much/very often to provide a total score. The scale has high internal reliability (alpha = .86) and was validated against diagnostic interviews, showing good agreement—if anything parents were more conservative with their rating of symptoms in a questionnaire than in a structured interview.

#### Childhood Autism Rating Scale (CARS)

The Childhood Autism Rating Scale (CARS) was developed to improve on previous classifications of children with autism. It is comprised of 15 scales measuring: Impairment in human relationships, Imitation, Inappropriate affect, Bizarre use of body movement and persistence of stereotypes, Peculiarities in relating to nonhuman objects, Resistance to environmental change, Peculiarities of visual responsiveness, Peculiarities of auditory responsiveness, Near receptor responsiveness, Anxiety reaction, Verbal communication, Nonverbal communication, Activity level, Intellectual functioning and General impressions. Each scale is scored on a continuum from normal (1) to severely abnormal (4), taking development for age level into consideration. These are summed to provide a total CARS score with a possible range from 15–60 whereby higher scores indicate higher degree of symptoms. The score has good internal consistency (reliability coefficient alpha = .94) and good construct validity as assessed by correlations with clinician ratings (*r* = .84, *P* < 0.001) [42]. In this study it was administered to the ASD sample.

### Fatty acid analysis

As part of the screening for nutrient deficiencies, clients were sent to Healthscope Pathology, a commercial Pathology Laboratory, for blood collection. Five milliliters of venous blood were collected in a Lithium Heparin tube, stored at room temperature and sent to the laboratory on the same day by courier, to be processed within two days of collection. Fatty acids were extracted from saline washed packed red cells, esterified and analysed by capillary gas chromatography, with flame ionization detection. Individual red cell fatty acids were reported as relative percentage of the total red cell fatty acids assayed [43]. Fatty acids that were available and investigated in these analyses were AA, EPA, DHA, AA/EPA ratio and total n-3/n-6 ratio.

### Statistical methods

Analyses were conducted using SPSS version 21. Means and standard deviations were computed and normal distribution assumptions were checked to ensure the validity of statistical tests. There were no missing data. Chi-square test was used to compare groups by gender composition. One-way analysis of variance (ANOVA) was performed with Tukey post-hoc analysis to compare groups with respect to age, test scores and PUFA levels. Pearson’s correlations were used to investigate associations between PUFA levels and scores for ADHD, TOVA and CARS. The significance level was set at the usual alpha = 5%.

## Results

**Fig 2:**
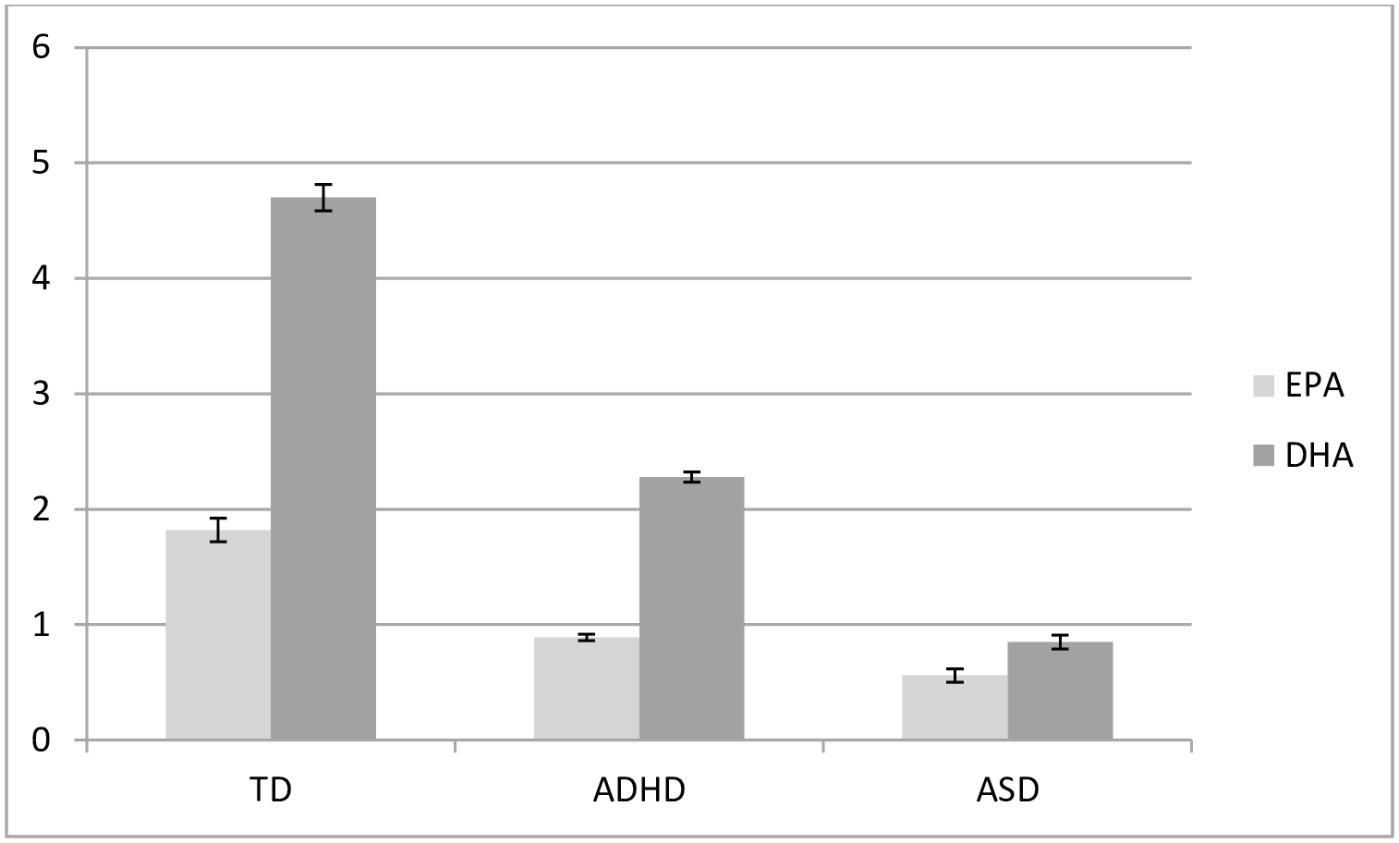
Erythrocyte EPA and DHA (as % of fatty acids) in children with ADHD (*n* = 401), ASD (*n* = 85) and typically developing (TD) controls (*n* = 79). Error bars represent standard error of the mean (SEM). All EPA and DHA comparisons are significantly different between groups (*P* < 0.001).

**Fig 3:**
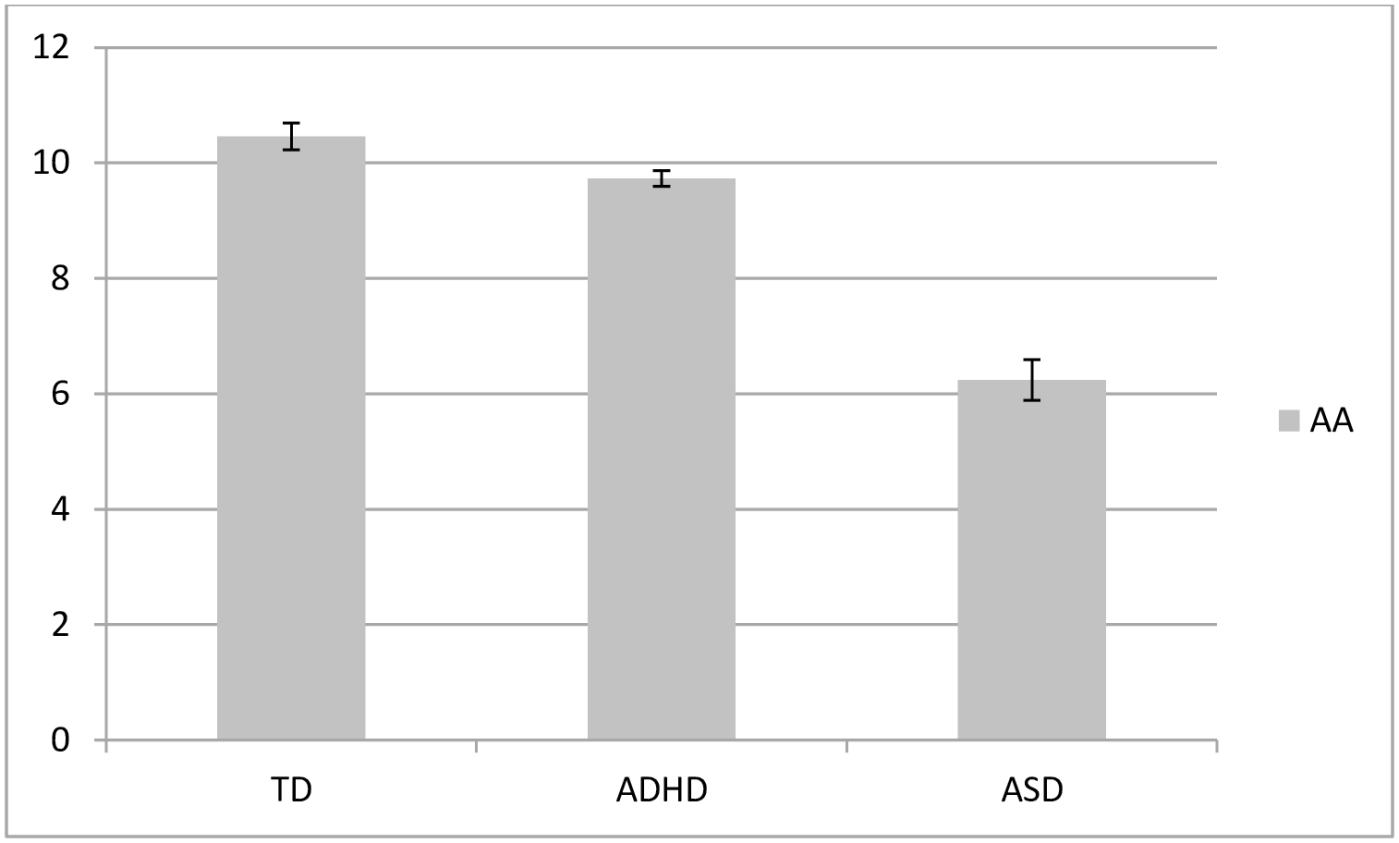
Erythrocyte AA (as % of fatty acids) in children with ADHD (*n* = 401), ASD (*n* = 85) and typically developing (TD) controls (*n* = 79). Error bars represent standard error of the mean (SEM). All are significantly different from each other (all *P* < 0.001 except TD vs ADHD: *P* = 0.047).

**Fig 4:**
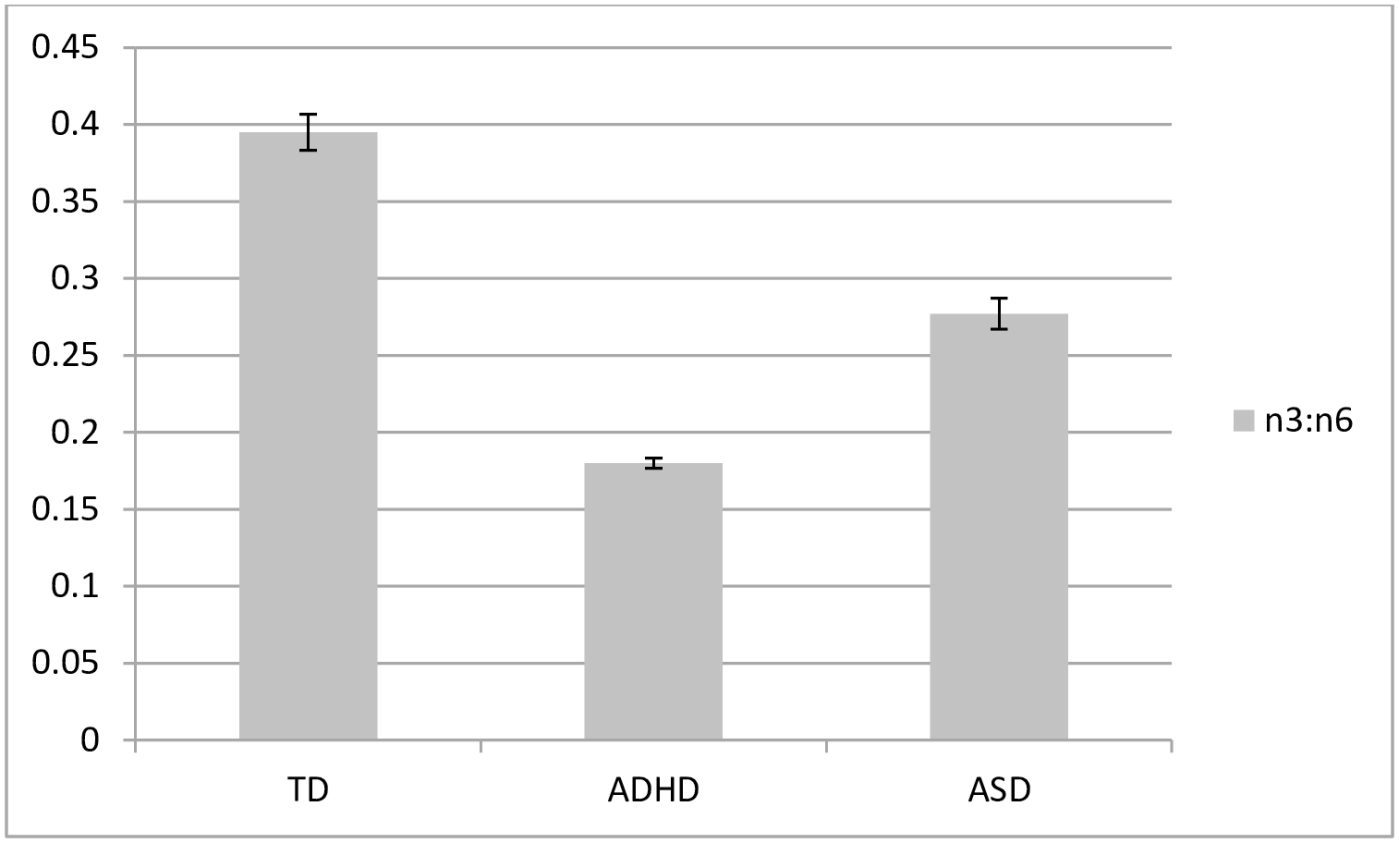
Erythrocyte omega-3 to omega-6 ratio (as % of fatty acids) in children with ADHD (*n* = 401), ASD (*n* = 85) and typically developing (TD) controls (*n* = 79). Error bars represent standard error of the mean (SEM). All comparisons are significantly different from each other (*P*<0.001).

Descriptive variables are presented in Table 1 including mean scores for the ADHD scale, TOVA and the CARS. As expected, children with ADHD had significantly higher scores on the ADHD scale and lower scores on the TOVA than controls. PUFA levels and comparisons between groups are provided in Table 2 and visually displayed in Figs 2–4. As predicted, children with ADHD and ASD had lower erythrocyte DHA, lower EPA, lower AA, higher AA/EPA and lower n-3/n-6 ratio than controls. Children with ASD had lower DHA, EPA and AA and higher n-3/n-6 ratio than children with ADHD.

**Table 2:** Erythrocyte PUFA levels in children with ADHD (*n* = 401), ASD (*n* = 85) and controls (*n* = 79) as % of fatty acids, and comparisons between groups using one-way ANOVA and Tukey’s HSD post-hoc analyses.

Variable	Group	M ± SD	Comparisons			
			Group	M diff	SE	*P*
EPA	ADHD	0.886 ± 0.563	ASD	0.329	0.073	0.000
			Control	-0.912	0.076	0.000
	ASD	0.557 ± 0.524	Control	-1.241	0.096	0.000
	Control	1.798 ± 0.894				
DHA	ADHD	2.281 ± 0.886	ASD	1.430	0.103	0.000
			Control	-2.434	0.107	0.000
	ASD	0.851 ± 0.564	Control	-3.865	0.135	0.000
	Control	4.715 ± 1.020				
AA	ADHD	9.726 ± 2.705	ASD	3.485	0.325	0.000
			Control	-0.796	0.335	0.047
	ASD	6.241 ± 3.277	Control	-4.280	0.425	0.000
	Control	10.522 ± 2.056				
EPA+DHA	ADHD	3.167 ± 1.254	ASD	1.760	0.148	0.000
			Control	-3.346	0.152	0.000
	ASD	1.407 ± 0.888	Control	-5.106	0.193	0.000
	Control	6.513 ± 1.446				
AA/EPA ratio	ADHD	14.314 ± 8.004	ASD	-0.354	0.925	0.922
			Control	6.524	0.954	0.000
	ASD	14.668 ± 8.534	Control	6.878	1.211	0.000
	Control	7.790 ± 5.053				
n-3/n-6 ratio	ADHD	0.180 ±0.065	ASD	-0.096	0.009	0.000
			Control	-0.215	0.009	0.000
	ASD	0.277 ± 0.093	Control	-0.118	0.012	0.000
	Control	0.395 ± 0.104				

**Fig 5:**
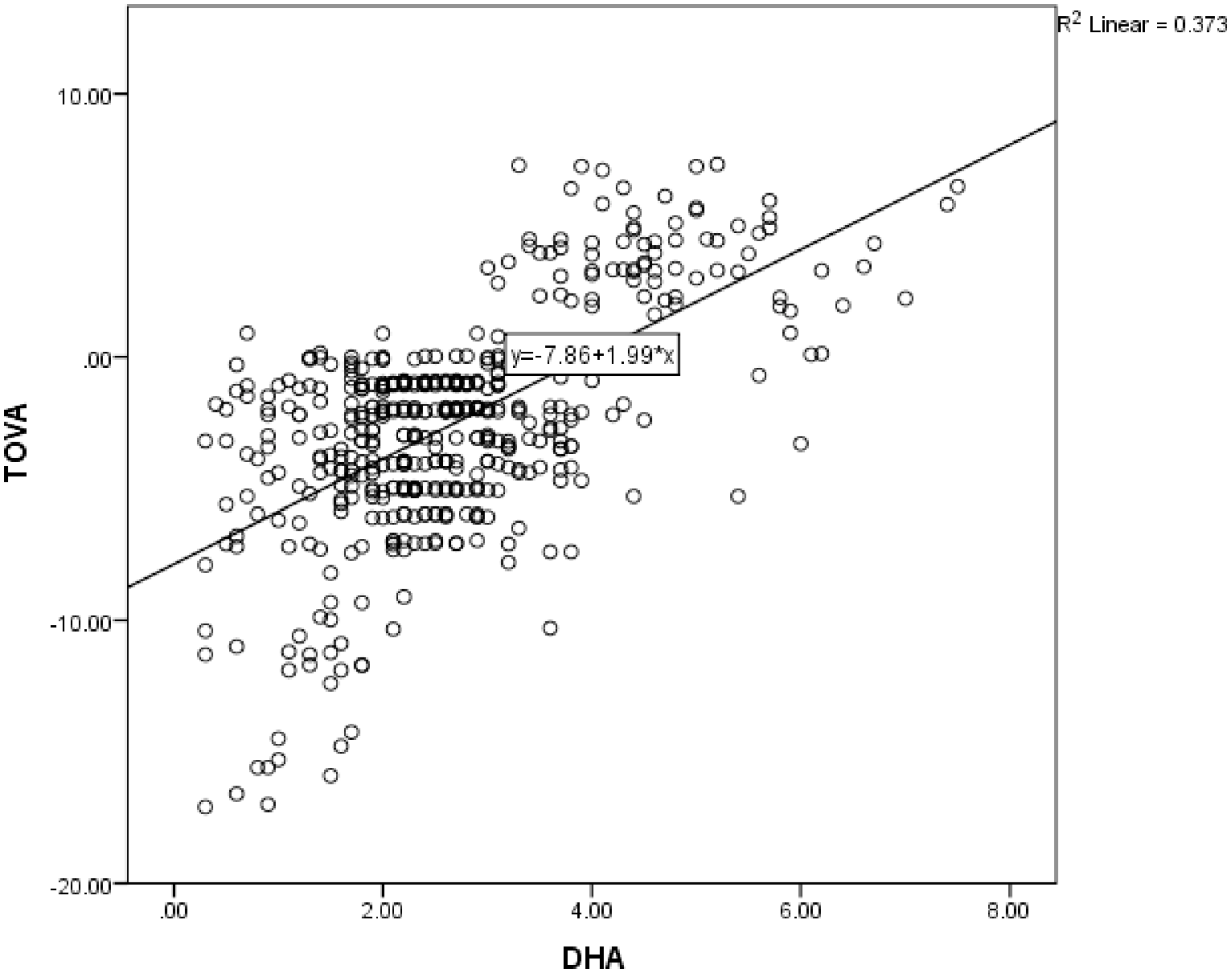
Scatterplot showing correlation between DHA and scores on the TOVA (Tests of Variable Attention; *r* = .610, *P* < 0.001; TOVA (Y) = 7.86–1.99 * DHA (X)) in children with ADHD and controls (*n* = 480). Note: higher TOVA scores = better attention.

**Fig 6:**
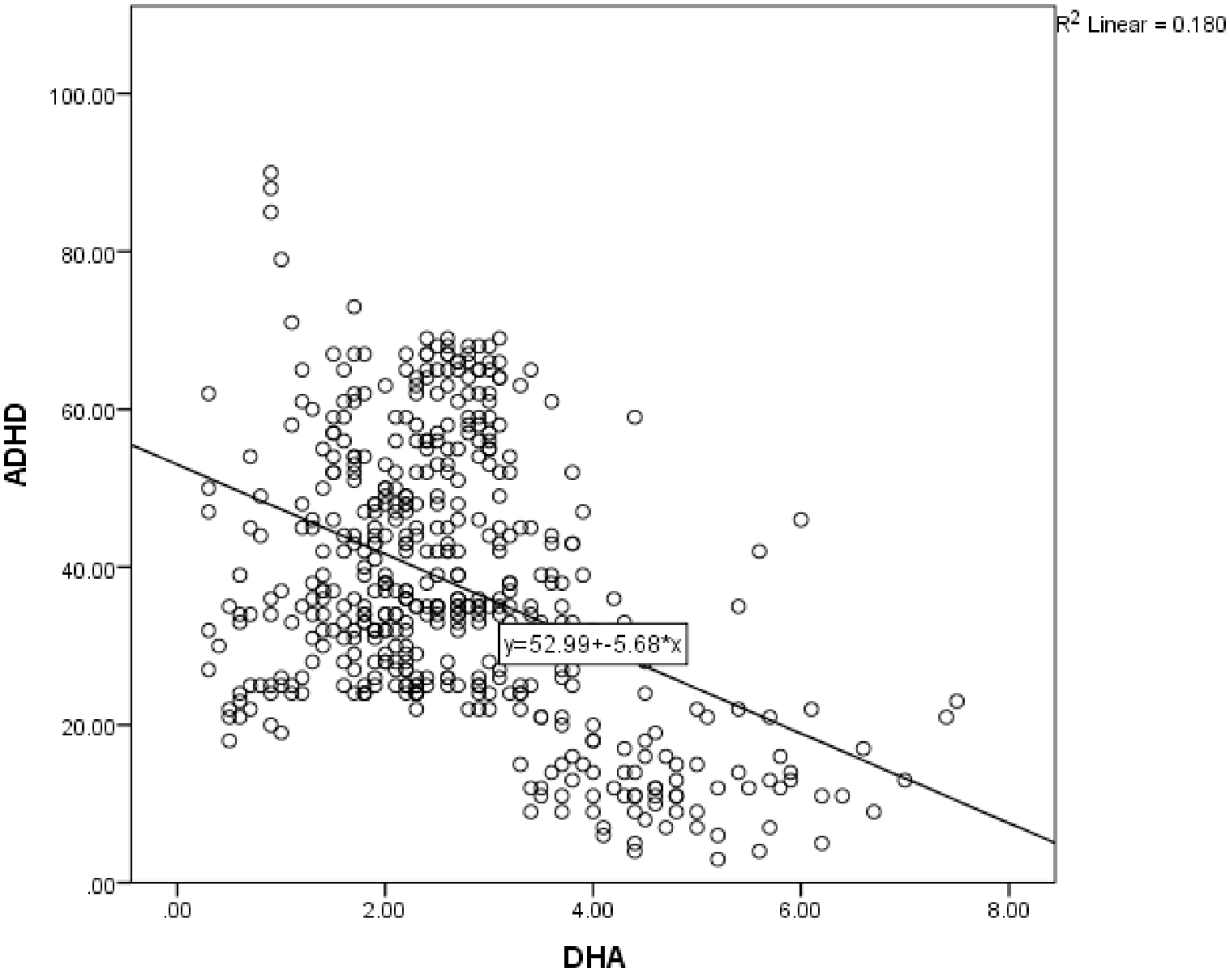
Scatterplot showing correlation between DHA and ADHD symptoms on the Australian Twin Behaviour Rating Scale (ATBRS; *r* = -.424; *P* < 0.001; ADHD (Y) = 52.99–5.68 * DHA (X)) in children with ADHD and controls (*n* = 480). Note: higher ADHD scores = greater severity of symptoms.

Correlations showed no significant relationship between age and scores on the ADHD/TOVA or CARS scales. Correlations between PUFAs and scores on the TOVA and CARS are shown in Table 3. As expected, lower DHA, EPA and ratio of n-3/n-6 PUFAs and higher ratio of AA/EPA were associated with higher ADHD scores in the sample of children with ADHD and controls, while higher DHA, EPA, AA and n3/n-6 ratio and lower AA/EPA ratio were associated with higher TOVA scores. Also consistent with hypotheses, in the children with ASD, lower EPA, DHA and AA were associated with higher CARS scores. Figs 5 and 6 show the scatterplots for the correlations between DHA and the TOVA and ADHD scores, respectively.

**Table 3:** Correlations between erythrocyte PUFAs and the TOVA (*n* = 480), ATBRS (*n* = 480), and CARS (*n* = 85).

	EPA	DHA	AA	EPA+DHA	n-3:n-6ratio	AA:EPAratio
TOVA	.418**	.610**	.199**	.604**	.509**	-.243**
ATBRS	-.294**	-.424**	-.003	-.421**	-.477**	.222**
CARS	-.255*	-.328**	-.251*	-.341**	.211	.029

** Correlation is significant at the *P* < 0.01 level (2-tailed)

* Correlation is significant at the *P* < 0.05 level (2-tailed)

TOVA = Test of Variable Attention (administered to children with ADHD and controls—higher scores represent better attention); ATBRS = Australian Twin Behaviour Rating Scale (measuring ADHD symptoms; administered to children with ADHD and controls—higher scores represent greater severity of symptoms); CARS = Childhood Autism Rating Scale (administered to children with ASD)

## Discussion

This study found lower levels of n-3 PUFAs EPA and DHA in children with ADHD and ASD compared with typically developing controls. As expected, lower levels of DHA, EPA and n-3/n-6 ratio and higher AA/EPA ratio were correlated with greater severity of symptoms. The lower levels of EPA and DHA are consistent with other reports [29, 34–38, 44]. Previous studies reported levels of erythrocyte DHA between 1.35% and 5.70% of cell membranes in children with ADHD compared with 2.08% to 5.83% for controls [30–32, 45–47] and when the studies were pooled, the mean difference between children with ADHD and controls was significant [29]. The pooled effect size was largest for DHA, suggesting that this PUFA was responsible for the overall difference in n-3 PUFA levels between children with ADHD and controls [29]. Levels of DHA in our study were 2.28% for children with ADHD, 0.85% for ASD and 4.72% for controls. The DOLAB study reported DHA levels of 1.9% and EPA 0.55% in UK school children who were underperforming in reading, although these samples were analysed in whole blood taken via finger prick so comparability may not be precise [48]. However they similarly reported correlations between lower DHA and poorer reading and ADHD-type behaviours. In children with autism, Bell et al. [36] reported DHA levels of 4.8% and 4.7% in children with classical autism/Asperger’s (*n* = 11) and regressive autism (*n* = 18), respectively, and 4.9% in non-aged matched controls (*n* = 55), which were not significantly different. However total n-3 PUFA and EPA levels were significantly lower in the autism groups compared with controls. Brigandi et al. reported DHA levels of 1.4% in erythrocyte membranes in children with autism compared to 1.76% in controls [49]. The DHA levels of 0.85% in our ASD sample is extremely low, particularly in comparison to 4.72% DHA in the control group.

According to the omega-3 index [50], levels of EPA + DHA under 4% confer greater risk of mortality from cardiovascular disease while levels of 8–12% provide the greatest protection. The omega-3 index in our study was 3.17% for children with ADHD and 1.41% for children with ASD compared with 6.51% for controls. Given that the omega-3 index is derived from erythrocyte membranes as a relative percentage of fatty acids rather than an absolute figure, it is unlikely to differ notably in children; therefore this is of concern for cardiovascular disease risk. A similar concern was raised by Montgomery et al. in relation to low omega-3 index levels in children from European countries [48]. Furthermore, although an omega-3 index for mental illness has not, to our knowledge, yet been established [44], these low levels may have significant implications also for mental health. It is possible that low n-3 PUFA levels contribute to common biological mechanisms underlying the psychiatric comorbidity between ADHD and ASD [51] and other psychiatric conditions [23, 28, 52].

The correlations between EPA and DHA and performance on the TOVA adds to a growing body of studies reporting improved sustained attention, particularly in relation to DHA, in ADHD [53–56]. Attention appears to be a core feature of neurodevelopmental disorders and may be a common factor in their high rate of comorbidities, particularly with learning disorders. Improved reading and ADHD-type symptoms have been found in children who do not have a diagnosis of ADHD following treatment with EPA plus DHA or DHA [57, 58]. Given that the diagnosis of ADHD is arbitrary, and symptoms occur on a continuum [41], these findings support a symptomatic rather than diagnostic approach to n-3 PUFA supplementation for learning and behaviour in children.

A number of studies in children with ADHD with and without comorbidities have reported improved symptoms with a supplement containing a 3/1 ratio of EPA/DHA (e.g. [58, 59, 60]), leading to conclusions in a meta-analysis that EPA has stronger effects than DHA [61]. However there are a number of methodological considerations that might have contributed to differences between studies, outlined elsewhere [24, 62]. Furthermore, these studies didn’t take blood samples so it is unclear the degree to which EPA and/or DHA contributed to improvements. Since this meta-analysis a study by Milte et al. reported lower DHA levels in children with ADHD and learning difficulties compared to children who did not have learning difficulties [63]. Children were then supplemented with high DHA, high EPA or omega-6 (linoleic acid) control and erythrocyte blood samples were taken [33, 64]. Results showed that increased EPA and DHA were associated with improved cognition and behaviour, and these associations were strongest for DHA. The DOLAB study gave school children whose reading performance was in the lowest 33^rd^ centile a pure DHA supplement and reported improved parent-rated ADHD-type symptoms and reading in children whose initial reading performance was in the lowest 20^th^ centile [57]. In the present study correlations between DHA and all psychological outcome measures were notably stronger than for EPA, also supporting the role of DHA in neurodevelopmental disorders. Future studies should therefore consider DHA supplementation combined with EPA and take blood samples where possible to further explore the differential benefits of these n-3 PUFAs.

Contrary to expectations, the present study found lower levels of AA in children with ADHD and particularly ASD compared with controls and small significant correlations between lower AA and poorer scores on the TOVA in children with ADHD and the CARS for children with ASD. However, we found that the ratio of AA to EPA (both eicosanoid-producing PUFAs [26, 28]) was higher in children with ADHD and ASD than controls. The AA/EPA ratio was associated with poorer outcomes for the ADHD and TOVA scores but not CARS scores. Other studies have reported higher AA/EPA or n-6/n-3 ratios in children with ADHD compared with controls [30–32, 45] even with inconsistencies in absolute AA values. The children with ASD had a higher total n-3/n-6 ratio than the children with ADHD, although lower than controls, which may be an artefact of their very low levels of both n-3 and n-6 PUFAs. We did not measure dietary PUFA intake in this study. In other studies there have been variable reports regarding dietary n-3 PUFA intake in children with ADHD; some report no difference whereas others report lower intake [29] and one reported higher intake compared with a national sample [65]. In children with autism it is generally thought that dietary intake is reduced due to restricted food preferences although there is limited evidence to support this [66]. A recent study also found lower levels of both DHA and AA in children with ASD. Importantly, they reported that AA metabolite prostaglandin E2 (PGE2) was higher compared to controls and suggested that the lower PUFA levels may be a result of abnormal lipid metabolism [49]. This observation supports previous suggestions that children with neurodevelopmental disorders may have a problem with PUFA metabolism such as increased oxidation of lipid membranes, decreased peroxisomal activity, or overactive removal of fatty acids from membranes by phospholipase A2 [35, 67, 68].

Another explanation could involve the influence of gut microbiota on PUFA uptake and metabolism. Dietary supplementation with *Bifidobacteria* has been shown to increase tissue levels of EPA and DHA in mice, and dietary supplementation with parent n-3 PUFA alpha-linolenic acid (ALA) combined with *Bifidobacterium breve* resulted in higher liver EPA and brain DHA levels [69]. Gut endothelial barrier integrity may also be enhanced by the PUFAs dihomo-g-linolenic acid (DGLA), AA, EPA and DHA [70]. This is particularly interesting in light of a recent study that randomised 75 infants to probiotics or placebo and reported at 13-year follow up zero cases of ADHD or Asperger syndrome in the probiotic group versus 17.1% cases in the placebo group. These authors further reported lower numbers of *Bifidobacterium* species bacteria in the faeces of children who developed the neurodevelopmental disorders than healthy children during the first 3 months of life and lower *Lactobacillus-Enterococcus* group bacteria and *Bacteroides* at 6 months [13]. Could some of this effect have been mediated by improved lipid profiles?

This cross-sectional study is the largest of its kind, supporting previous work that showed low n-3 PUFA levels, particularly DHA, in children with neurodevelopmental disorders. Furthermore, our study shows strong correlations with symptoms. Results are likely to be more generalisable than the few smaller studies that have been conducted, and the low levels of both DHA and AA in children with ASD supports a recent, larger study in this population [49]. We are unable to determine from our data whether children with neurodevelopmental disorders have poorer diets leading to low n-3 PUFAs and high n-6/n-3 PUFA ratio or whether these are contributing to their symptoms. The structural and functional roles of PUFAs in neuronal membranes support underlying biological mechanisms and a growing body of research evidence suggests that supplementation can assist with symptoms in children with ADHD [24] and other psychiatric disorders [62]. Future work should further explore fatty acid metabolism in neurodevelopmental disorders, the role of the gut microbiota, and predictors of response to treatment. Altered fatty acid metabolism may be particularly pertinent to children with ASD; the low levels of both n-3 and n-6 PUFA in these children require further exploration.

## References

[1] Pliszka SR. Comorbidity of attention-deficit/hyperactivity disorder with psychiatric disorder: An overview. J Clin Psychiatry. 1998;59(suppl 7):50–8. 9680053

[2] Simonoff E, Pickles A, Charman T, Chandler S, Loucas T, Baird G. Psychiatry disorders in children with austism spectrum disorders: prevalence, comorbidity, and associated factors in a population-derived sample. J Am Acad Child Adolesc Psychiatry. 2008;47(8):921–9. 10.1097/CHI.0b013e318179964f 18645422

[3] Polanczyk G, de Lima MS, Horta BL, Biederman J, Rohde LA. The worldwide prevalence of ADHD: A systematic review and metaregression analysis. Am J Psychiatry. 2007;164(6):942–8. 17541055 10.1176/ajp.2007.164.6.942

[4] Zablotsky B, Black LI, Maenner MJ, Scheive LA, Blumberg SJ. National Health Statistics Reports. US Department of Health and Human Services, 2013.

[5] Muhle R, Trentacoste SV, Rapin IR. The genetics of autism. Pediatrics. 2004;113(5):e472–e86. 15121991 10.1542/peds.113.5.e472

[6] Nikolas M, Burt AS. Genetic and environmental influences on ADHD symptom dimensions of inattention and hyperactivity: A meta-analysis. J Abnorm Psychol. 2010;119(1):1–17. 10.1037/a0018010 20141238

[7] Grandjean P, Landrigan PJ. Neurobehavioural effects of developmental toxicity. The Lancet Neurol. 2014;13(3):330–8. 10.1016/S1474-4422(13)70278-3 24556010 PMC4418502

[8] Miller CS. The compelling anomaly of chemical intolerance. Ann N Y Acad Sci. 2001;933(1):1–23. 12000012 10.1111/j.1749-6632.2001.tb05810.x

[9] Howard AL, Robinson M, Smith GJ, Ambrosini GL, Piek JP, Oddy WH. ADHD is associated with a "Western" dietary pattern in adolescents. J Atten Disord. 2011;15(5):403–11. 10.1177/1087054710365990 20631199

[10] Kohlboeck G, Sausenthaler S, Standl M, Koletzko S, Bauer C-P, von Berg A, et al Food intake, diet quality and behavioral problems in children: results from the GINI-plus/LISA-plus studies. Ann Nutr Metab. 2012;60:247–56. 10.1159/000337552 22677949

[11] Wiles NJ, Northstone K, Emmett P, Lewis G. 'Junk food' diet and childhood behavioural problems: results from the ALSPAC cohort. Eur J Clin Nutr. 2007;63:491–8. 18059416 10.1038/sj.ejcn.1602967 PMC2664919

[12] Rowland AS, Lesesne CA, Abramowitz AJ. The epidemiology of attention-deficit/hyperactivity disorder (ADHD): A public health view. Ment Retard Dev Disabil Res Rev. 2002;8(3):162–70. 12216060 10.1002/mrdd.10036

[13] Pärtty A, Kalliomaki M, Wacklin P, Salminen S, Isolauri E. A possible link between early probiotic intervention and the risk of neuropsychiatric disorders later in childhood: a randomized trial. Pediatr Res. 2015;77(6):823–8. 10.1038/pr.2015.51 25760553

[14] American Psychiatric Association. Diagnostic and Statistical Manual of Mental Disorders (DSM-5). Arlington, Va: American Psychiatric Association; 2013.

[15] Charach A, Ickowicz A, Schachar R. Stimulant treatment over five years: adherence, effectiveness, and adverse effects. Journal of the American Academy of Child & Adolescent Psychiatry. 2004;43(5):559–67. 15100562 10.1097/00004583-200405000-00009

[16] Jensen PS, Arnold EL, Swanson JM, Vitiello B, Abikoff HB, Greenhill LL, et al 3-year follow-up of the NIMH MTA Study. J Am Acad Child Adolesc Psychiatry. 2007;46(8):989–1002. 17667478 10.1097/CHI.0b013e3180686d48

[17] Molina BSG, Flory K, Hinshaw SP, Greiner AR, Arnold LE, Swanson JM, et al Delinquent behavior and emerging substance use in the MTA at 36 months: prevalence, course, and treatment effects. J Am Acad Child Adolesc Psychiatry. 2007;46(8):1028–40. 17667481 10.1097/chi.0b013e3180686d96

[18] MTA Cooperative Group. National Institute of Mental Health multimodal treatment study of ADHD follow-up: changes in effectiveness and growth after the end of treatment. Pediatrics. 2004;113:762–9. 15060225 10.1542/peds.113.4.762

[19] Swanson JM, Elliott GR, Greenhill LL, Wigal T, Eugene AL, Vitiello B, et al Effects of Stimulant Medication on Growth Rates Across 3 Years in the MTA Follow-up. J Am Acad Child Adolesc Psychiatry. 2007;46(8):1015–27. 17667480 10.1097/chi.0b013e3180686d7e

[20] Curtis LT, Patel K. Nutritional and environmental approaches to preventing and treating autism and attention deficit hyperactivity disorder (ADHD): A review. J Altern Complement Med. 2008;14(1):79–85. 10.1089/acm.2007.0610 18199019

[21] Transler C, Eilander A, Mitchell S, van de Meer N. The impact of polyunsaturated fatty acids in reducing child attention deficit and hyperactivity disorders. J Atten Disord. 2010;14(3):232–46. 10.1177/1087054709347250 20424008

[22] Sinn N. Nutritional and dietary influences on attention deficit hyperactivity disorder. Nutr Rev. 2008;66(10):558–68. 10.1111/j.1753-4887.2008.00107.x 18826452

[23] Sinn N, Wilson C. Dietary supplementation with highly unsaturated fatty acids: Implications for interventions with persons with mental retardation from research on infant cognitive development, ADHD, and other developmental disabilities. Int Rev Res Ment Retard. 2006;32:159–96.

[24] Gow RV, Hibbeln JR, Parletta N. Current evidence and future directions for research with omega-3 fatty acids and attention deficit hyperactivity disorder. Curr Opin Clin Nutr Metab Care. 2015;18(2):133–8. 10.1097/MCO.0000000000000140 25581035

[25] Parletta N, Milte CM, Meyer B. Nutritional modulation of cognitive function and mental health. J Nutr Biochem. 2013;24(5):725–43. 10.1016/j.jnutbio.2013.01.002 23517914

[26] Simopoulos AP. The importance of the omega-6/omega-3 fatty acid ratio in cardiovascular disease and other chronic diseases. Exp Biol Med. 2008;233(6):674–88. 10.3181/0711-MR-311 18408140

[27] Simopoulos AP. The importance of the ratio of omega-6/omega-3 essential fatty acids. Biomed Pharmacother. 2002;56(8):365–79. 12442909 10.1016/s0753-3322(02)00253-6

[28] Sinn N, Howe PRC. Mental health benefits of omega-3 fatty acids may be mediated by improvements in cerebral vascular function. Biosci Hypotheses. 2008;1(2):103–8.

[29] Hawkey E, Nigg JT. Omega-3 fatty acid and ADHD: Blood level analysis and meta-analytic extension of supplementation trials. Clin Psychol Rev. 2014;34(6):496–505. 10.1016/j.cpr.2014.05.005 25181335 PMC4321799

[30] Antalis CJ, Stevens LJ, Campbell M, Pazdro R, Ericson K, Burgess JR. Omega-3 fatty acid status in attention-deficit/hyperactivity disorder. Prostaglandins Leukot Essent Fatty Acids. 2006;75:299–308. 16962757 10.1016/j.plefa.2006.07.004

[31] Chen J-R, Hsu S-F, Hsu C-D, Hwang L-H, Yang S-C. Dietary patterns and blood fatty acid composition in children with attention-deficit hyperactivity disorder in Taiwan. J Nutr Biochem. 2004;15:467–72. 15302081 10.1016/j.jnutbio.2004.01.008

[32] Laasonen M, Hokkanen L, Leppämäki S, Tani P, Erkkilä AT. Project DyAdd: Fatty acids and cognition in adults with dyslexia, ADHD, or both. Prostaglandins Leukot Essent Fatty Acids. 2009;81:79–88. 10.1016/j.plefa.2009.04.004 19464861

[33] Milte CM, Parletta N, Buckley J, Coates A, Young R, Howe P. Increased erythrocyte eicosapentaenoic acid and docosahexaenoic acid are associated with improved attention and behaviour in children with ADHD in a 12-month randomised controlled three-way crossover trial. J Atten Disord. 2015;19(11):954–964. 10.1177/1087054713510562 24214970

[34] Vancassel S, Durand G, Barthelemy C. Plasma fatty acid levels in autistic children. Prostaglandins Leukot Essent Fatty Acids. 2001;65:1–7. 11487301 10.1054/plef.2001.0281

[35] Wiest MM, German JB, Harvey DJ, Watkins SM, Hertz-Picciotto I. Plasma fatty acid profiles in autism: A case-control study. Prostaglandins Leukot Essent Fatty Acids. 2009;80(4):221–7. 10.1016/j.plefa.2009.01.007 19307110

[36] Bell JG, MacKinlay EE, Dick JR, MacDonald DJ, Boyle RM, Glen ACA. Essential fatty acids and phospholipase A2 in autistic spectrum disorders. Prostaglandins Leukot Essent Fatty Acids. 2004;71:201–4. 15301788 10.1016/j.plefa.2004.03.008

[37] Bell JG, Sargent JR, Tocher DR, Dick JR. Red blood cell fatty acid compositions in a patient with autistic spectrum disorder: a characteristic abnormality in neurodevelopmental disorders? Prostaglandins Leukot Essent Fatty Acids. 2000;63(2/1):21–5. 10970708 10.1054/plef.2000.0186

[38] Bu B, Ashwood P, Harvey D, King IB, Van de Water J, Jin L-W. Fatty acid compositions of red blood cell phospholipids in children with autism. Prostaglandins Leukot Essent Fatty Acids. 2006;74(4):215–21. 16581239 10.1016/j.plefa.2006.02.001

[39] Greenberg LM, Waldman ID. Developmental normative data on the Test of Variables of Attention (TOVA). J Child Adolesc Psychiatry. 1993;34:1019–30. 10.1111/j.1469-7610.1993.tb01105.x 8408366

[40] Llorente AM, Voigt R, Jensen CL, Fraley JK, Heird WC, Rennie KM. The Test of Variables of Attention (TOVA): Internal consistency (Q1 vs. Q2 and Q3 vs. Q4) in children with Attention Deficit/Hyperactivity Disorder (ADHD). Child Neuropsychol. 2008;14(4):314–22. 10.1080/09297040701563578 17917866

[41] Levy F, Hay D, McStephen M, Wood C, Waldman I. Attention-deficit hyperactivity disorder: a category or a continuum? Genetic analysis of a large-scale twin study. J Am Acad Child Adolesc Psychiatry. 1997;36(6):737–44. 9183127 10.1097/00004583-199706000-00009

[42] Schopler E, Reichler RJ, DeVellis RF, Daly K. Toward objective classification of childhood autism: Childhood Autism Rating Scale (CARS). J Autism Dev Disord. 1980;10(1):91–103. 6927682 10.1007/BF02408436

[43] Shamberger RJ. Erythrocyte fatty acid studies in patients. J Adv Med. 1997;10(3):195–205.

[44] Milte C, Sinn N, Howe PRC. Polyunsaturated fatty acid status in ADHD, depression and dementia: towards an omega-3 index for mental health? Nutr Rev. 2009;67(10):573–90. 10.1111/j.1753-4887.2009.00229.x 19785689

[45] Colter AL, Cutler C, Meckling KA. Fatty acid status and behavioural symptoms of attention deficit hyperactivity disorder in adolescents: a case control study. Nutr J. 2008;7:8 10.1186/1475-2891-7-8 18275609 PMC2275745

[46] Joshi K, Lad S, Kale M, Patwardhan B, Mahadik SP, Patni B, et al Supplementation with flax oil and vitamin C improves the outcome of attention deficity hyperactivity disorder (ADHD). Prostaglandins Leukot Essent Fatty Acids. 2006;74:17–21. 16314082 10.1016/j.plefa.2005.10.001

[47] Young G, Maharaj NJ, Conquer J. Blood phospholipid fatty acid analysis of adults with and without attention deficit/hyperactivity disorder. Lipids. 2004;39:117–23. 15134138 10.1007/s11745-004-1209-3

[48] Montgomery P, Burton JR, Sewell RP, Spreckelsen TF, Richardson AJ. Low blood long chain omega-3 fatty acids in UK children are associated with poor cognitive performance and behavior: A cross-sectional analysis from the DOLAB study. PLoS ONE. 2013;8(6):e66697 10.1371/journal.pone.0066697 23826114 PMC3691187

[49] Brigandi SA, Shao H, Qian SY, Shen Y, Wu B-L, Kang JX. Autistic children exhibit decreased levels of essential fatty acids in red blood cells. Int J Mol Sci. 2015;16(5):10061–76. 10.3390/ijms160510061 25946342 PMC4463632

[50] Harris WS, von Schacky C. The Omega-3 Index: a new risk factor for death from coronary heart disease? Prev Med. 2004;39:212–20. 15208005 10.1016/j.ypmed.2004.02.030

[51] Reiersen AM, Todd RD. Co-occurrence of ADHD and autism spectrum disorders: phenomenology and treatment. Expert Review Neurother. 2008;8(4):657–69. 10.1586/14737175.8.4.657 18416666

[52] Richardson AJ. Long-chain polyunsaturated fatty acids in childhood develpomental and psychiatric disorders. Lipids. 2004;39(12):1215–22. 15736918 10.1007/s11745-004-1350-z

[53] Bos DJ, Oranje B, Veerhoek ES, Van Diepen RM, Weusten JM, Demmelmair H, et al Reduced Symptoms of Inattention after Dietary Omega-3 Fatty Acid Supplementation in Boys with and without Attention Deficit/Hyperactivity Disorder. Neuropsychopharmacol. 2015. 10.1038/npp.2015.73 PMC4538345 25790022

[54] McNamara RK, Able J, Jandacek R, Rider T, Tso P, Eliassen JC, et al Docosahexaenoic acid supplementation increases prefrontal cortex activation during sustained attention in healthy boys: a placebo-controlled, dose-ranging, functional magnetic resonance imaging study. Am J Clin Nutr. 2010;91:1060–7. 10.3945/ajcn.2009.28549 20130094 PMC2844685

[55] Vaisman N, Kaysar N, Zaruk-Adasha Y, Pelled D, Brichon G, Zwingelstein G, et al Corelation between changes in blood fatty acid composition and visual sustained attention performance in children with inattention: effect of dietary n-3 fatty acids containing phospholipids. Am J Clin Nutr. 2008;87:1170–80. 18469236 10.1093/ajcn/87.5.1170

[56] Sinn N, Bryan J, Wilson C. Cognitive effects of polyunsaturated fatty acids in children with attention deficit hyperactivity disorder symptoms: A randomised controlled trial. Prostaglandins LeukotEssent Fatty Acids. 2008;78(4–5):311–26. 10.1016/j.plefa.2008.04.004 18514501

[57] Richardson AJ, Burton JR, Sewell RP, Montgomery P. Docosahexaenoic acid for reading, cognition and behaviour in children aged 7–9 years: A randomised, controlled trial (the DOLAB study). PLoS ONE. 2012;7(9):E43909 10.1371/journal.pone.0043909 22970149 PMC3435388

[58] Richardson AJ, Montgomery P. The Oxford-Durham study: a randomised, controlled trial of dietary supplementation with fatty acids in children with developmental coordination disorder. Pediatrics. 2005;115:1360–6. 15867048 10.1542/peds.2004-2164

[59] Johnson M, Östlund S, Fransson G, Kadesjö B, Gillberg C. Omega-3/omega-6 fatty acids for attention deficit hyperactivity disorder. J Atten Disord. 2009;12(5):394–401. 10.1177/1087054708316261 18448859

[60] Sinn N, Bryan J. Effect of supplementation with polyunsaturated fatty acids and micronutrients on ADHD-related problems with attention and behavior. J Dev Behav Pediatr. 2007;28(2):82–91. 17435458 10.1097/01.DBP.0000267558.88457.a5

[61] Bloch MH, Qawasmi A. Omega-3 fatty acid supplementation for the treatment of children with attention-deficit/hyperactivity disorder symptomatology: Systematic review and meta-analysis. J Am Acad Child Adolesc Psychiatry. 2011;50(10):991–1000. 10.1016/j.jaac.2011.06.008 21961774 PMC3625948

[62] Sinn N, Milte C, Howe PRC. Oiling the brain: A review of randomised controlled trials of omega-3 fatty acids in psychopathology across the lifespan. Nutrients. 2010;2(2):128–70. 10.3390/nu2020128 22254013 PMC3257637

[63] Milte C, Sinn N, Buckley JD, Coates AM, Young RM, Howe PRC. Erythrocyte polyunsaturated fatty acids, cognition and literacy in children with ADHD with and without learning difficulties. J Child Health Care. 2011;15(4):299–311. 10.1177/1367493511403953 21828168

[64] Milte CM, Parletta N, Buckley JD, Coates AM, Young RM, Howe PRC. Eicosapentaenoic and docosahexaenoic acids, cognition, and behavior in children with attention deficit hyperactivity disorder: A randomized controlled trial. Nutrition. 2012;28(6):670–7. 10.1016/j.nut.2011.12.009 22541055

[65] Ng K-H, Meyer B, Reece L, Sinn N. Dietary polyunsaturated fatty acid intakes in children with ADHD symptoms. Br J Nutr. 2009;102:1635–41. 10.1017/S0007114509990821 19631022

[66] Johnson CR, Handen BL, Mayer-Costa M, Sacco K. Eating habits and dietary status in young children with autism. J Dev Phys Disabil. 2008;20(5):437–48.

[67] Ming X, Stein TP, Brimacombe M, Johnson WG, Lambert GH, Wagner GC. Increased excretion of a lipid peroxidation biomarker in autism. Prostaglandins Leukot Essent Fatty Acids. 2005;73:379–84. 16081262 10.1016/j.plefa.2005.06.002

[68] Richardson AJ, Ross MA. Fatty acid metabolism in neurodevelopmental disorder: a new perspective on associations between attention-deficit/hyperactivity disorder, dyslexia, dyspraxia and the autistic spectrum. Prostaglandins Leukot Essent Fatty Acids. 2000;63(1/2):1–9. 10970706 10.1054/plef.2000.0184

[69] Wall R, Marques TM, O'Sullivan O, Ross RP, Shanahan F, Quigley EM, et al Contrasting effects of *Bifidobacterium breve* NCIMB 702258 and *Bifidobacterium breve* DPC 6330 on the composition of murine brain fatty acids and gut microbiota. Am J Clin Nutr. 2012;95:1278–87. 10.3945/ajcn.111.026435 22492373

[70] Willemsen LE, Koetsier MA, Balvers M, Beermann C, Stahl B, van Tol EAF. Polyunsaturated fatty acids support epithelial barrier integrity and reduce IL-4 mediated permeability in vitro. Eur J Nutr. 2008;47(4):183–91. 10.1007/s00394-008-0712-0 18497998

